# Cooperation through Competition—Dynamics and Microeconomics of a Minimal Nutrient Trade System in Arbuscular Mycorrhizal Symbiosis

**DOI:** 10.3389/fpls.2016.00912

**Published:** 2016-06-27

**Authors:** Stephan Schott, Braulio Valdebenito, Daniel Bustos, Judith L. Gomez-Porras, Tripti Sharma, Ingo Dreyer

**Affiliations:** Facultad de Ingeniería, Centro de Bioinformática y Simulación Molecular, Universidad de TalcaTalca, Chile

**Keywords:** computational cell biology, phosphate, plant biophysics, proton-coupled transport, sugar

## Abstract

In arbuscular mycorrhizal (AM) symbiosis, fungi and plants exchange nutrients (sugars and phosphate, for instance) for reciprocal benefit. Until now it is not clear how this nutrient exchange system works. Here, we used computational cell biology to simulate the dynamics of a network of proton pumps and proton-coupled transporters that are upregulated during AM formation. We show that this minimal network is sufficient to describe accurately and realistically the nutrient trade system. By applying basic principles of microeconomics, we link the biophysics of transmembrane nutrient transport with the ecology of organismic interactions and straightforwardly explain macroscopic scenarios of the relations between plant and AM fungus. This computational cell biology study allows drawing far reaching hypotheses about the mechanism and the regulation of nutrient exchange and proposes that the “cooperation” between plant and fungus can be in fact the result of a competition between both for the same resources in the tiny periarbuscular space. The minimal model presented here may serve as benchmark to evaluate in future the performance of more complex models of AM nutrient exchange. As a first step toward this goal, we included SWEET sugar transporters in the model and show that their co-occurrence with proton-coupled sugar transporters results in a futile carbon cycle at the plant plasma membrane proposing that two different pathways for the same substrate should not be active at the same time.

## Introduction

Land plants established diverse forms of mutualistic and reciprocally beneficial symbiotic relationships with microorganisms (Marschner, 2012). In the most prevalent symbiosis known, arbuscular mycorrhizal fungi (AMF) from the phylum Glomeromycota colonize the root systems and modulate plant growth by enhancing the availability of nutrients (Parniske, 2008; Bonfante and Genre, 2010; Smith and Smith, 2011; Harrison, 2012; van der Heijden et al., 2015). Here, the plant benefits in particular from the phosphorus supply by the fungus and provides the fungus in return with energy-rich photosynthetic carbohydrates (Karandashov and Bucher, 2005; Kiers et al., 2011; Smith et al., 2011). The fungus with its extensive network of extra-radical hyphae extends beyond root depletion zones and can explore larger soil volumes; it thus secures new regions for nutrient mining.

When a hypha from a germinating soil-borne spore of an AM fungus comes into contact with a host root, it starts to penetrate deep into the parenchyma cortex and then differentiates into highly branched arbuscular structures. As a response, the infected cells undergo major modifications by altering their transcriptional activity, shrinking the vacuoles, reorganizing the cytoskeleton and extending the plant plasma membrane that it surrounds the arbuscule (Gianinazzi-Pearson, 1996; Harrison, 2012). The fractal-like structure of fungal and plant plasma membranes significantly increases the interaction surface between fungus and plant. The symbiotic interface, the periarbuscular space, separates the two plasma membranes by 100 nm or less and is a specialized apoplastic zone for nutrient exchange between the mycorrhizal symbionts (Gianinazzi-Pearson, 1996).

Along with the morphological changes, gene expression is also largely re-programmed in arbuscule containing cells (Harrison, 2012). In particular, the expression of certain types of transmembrane transporters is stimulated. So far it was known that at the plant side, genes coding for H^+^-ATPases, proton-coupled sugar transporters, and proton-coupled phosphate transporters are activated in arbuscule containing cells (Gianinazzi-Pearson, 1996; Harrison, 1996, 2012; Rausch et al., 2001; Harrison et al., 2002; Paszkowski et al., 2002; Javot et al., 2007; Krajinski et al., 2014), while at the fungal side, this transporter set is complemented by proton pumps as well as sugar and phosphate transporters that are homologous to the plant transporters (Harrison and van Buuren, 1995; Karandashov and Bucher, 2005; Ramos et al., 2008; Helber et al., 2011; Doidy et al., 2012). Very recently it was shown that in plant cells also carbon transporters of the SWEET type are upregulated during arbuscule formation (Manck-Götzenberger and Requena, 2016).

The exchange of phosphate (P) for carbon (C) is seen as a highly cooperative process. Plants can detect, discriminate, and reward the best fungal partners with more carbohydrates. In turn, their fungal partners enforce cooperation by increasing nutrient transfer only to those roots providing more carbohydrates (Kiers et al., 2011). This exchange of nutrients has been considered as an ideal example to test biological market theory (Noë and Hammerstein, 1995; Schwartz and Hoeksema, 1998; Hoeksema and Schwartz, 2003) and was addressed by Kiers et al. (2011) proposing that AMF symbiosis functions analogous to a market economy, where there are partners on both sides of the interaction and higher quality services are remunerated in both directions. However, the terms of trade between the partners are still under debate (Fitter, 2006; Smith and Smith, 2015). In particular, the molecular components involved in the efflux of P across the fungal plasma membrane and in the export of carbohydrates from the plant have not been clarified yet (Bonfante and Genre, 2010; Smith and Smith, 2011; Johri et al., 2015).

Interestingly both, plant and fungus, are equipped with electrically identical transporters: (i) proton pumps, (ii) H^+^/sugar transporters that commonly use the electrochemical proton gradient for the uptake of sugars (Klepek et al., 2005), and (iii) phosphate transporters with a stoichiometry of *n*H^+^:Pi^−^ (*n* > 1) that normally harvest the electrochemical proton gradient for the uptake of phosphate (Preuss et al., 2011). In principle, these transporter types might be sufficient for nutrient exchange as proton-coupled transporters are not only suited for the uptake of nutrients but also for their release; a transport mode that is often overseen. They work as perfect molecular machines that transport their substrate(s) along the coupled electrochemical gradients without rectification preferences (Carpaneto et al., 2005; Preuss et al., 2011).

To test whether the known proton-coupled transporters are sufficient for the nutrient exchange between plant and fungus we simulated in this study the transporter network in the AM interaction zone *in silico*. We show that a network of these transporters, which have been considered so far to mediate sugar or phosphate uptake only, functions in fact as a nutrient trade system with all properties that are observed in AM symbiosis. The presented computational cell biological data allow insights that are far beyond the reach of any wet-lab experimental technique available at the moment. The data suggest particular market forces of the P-C-exchange between plant and AM fungus, where their “cooperation” is the result of a competition of both for the same resources in the periarbuscular space. On the basis of this minimal model we evaluate the performance of the nutrient trade system if additionally sugar channels of the SWEET type are included.

## Materials and methods

### Geometry of the plant fungus interface

**Figure 1:**
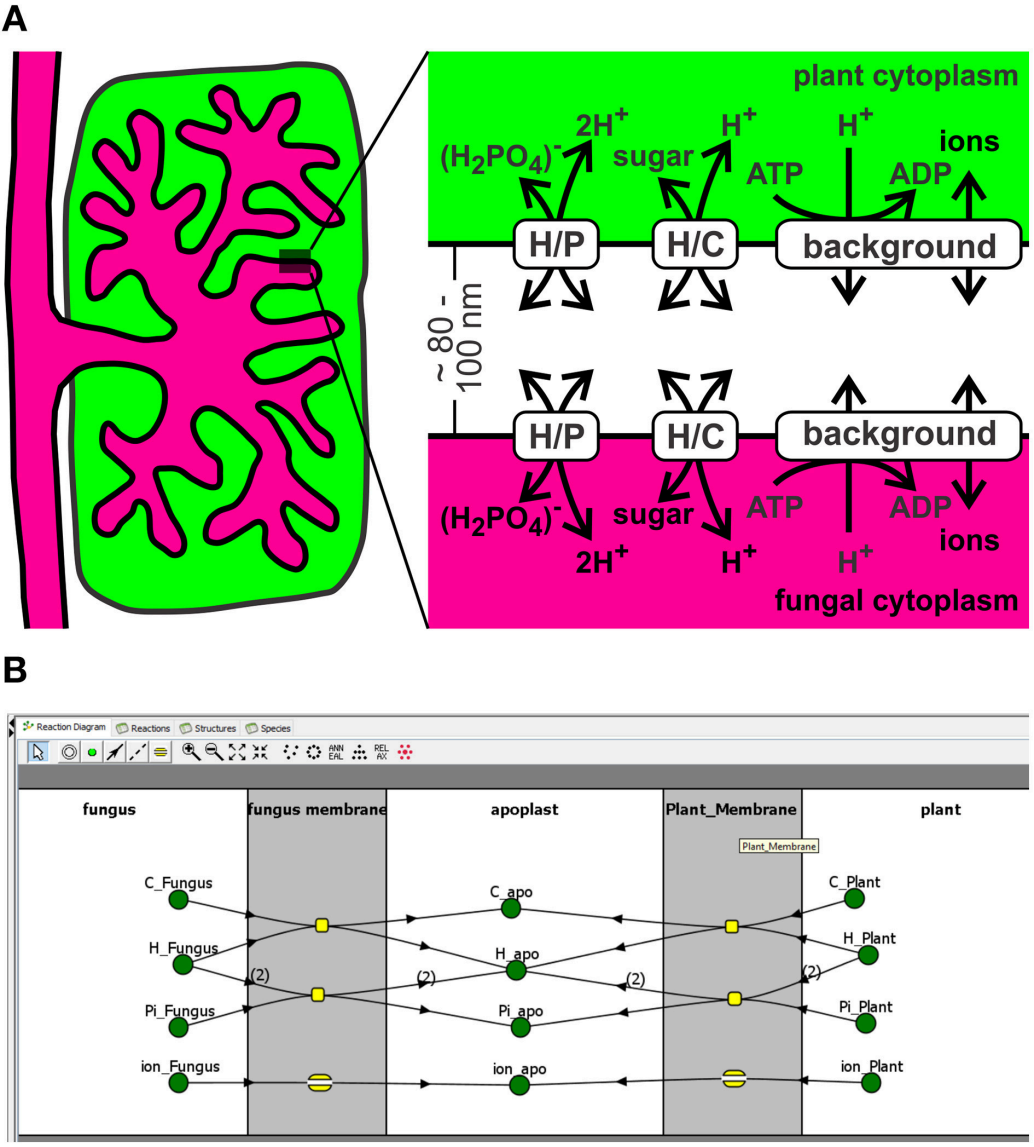
**A minimal transporter network for nutrient exchange in arbuscular mycorrhizal symbiosis. (A)** The exchange zone was modeled as a three-compartment system with the 100 nm thick interfacial apoplast between plant and fungus and 50-fold larger cytoplasmic volumes. Note that different cytoplasms/apoplasm values do not qualitatively change the obtained results. The pH values were kept constant to pHcyt = 7.0 and pHapo = 6.0 to reflect the proton-buffer capacities (see also Discussion for the flexibility of these values). Transport of sugar or phosphate was mediated by H^+^/sugar (H/C) and H^+^/phosphate (H/P) cotransporters. The background conductance gathers the H^+^-ATPase and other potential ionic conductances. In order to include the exchange of nitrogen sources, the network could be enlarged by ${\text{NH}}_{4}^{+}$-channels and electrogenic H^+^/${\text{NO}}_{3}^{-}$, H^+^/amino acid or H^+^/peptide co-transporters (Smith and Smith, 2011; Casieri et al., 2013). The qualitative conclusions would be identical to the considered P/C-exchange system. **(B)** Screenshot of the implementation of the three compartment model in VCell.

A small sector of the interaction zone at an arbuscule can be approximated by two parallel plasma membranes separated by ~80–100 nm (Figure 1A). In this three compartment model the plant cytoplasm and the fungal cytoplasm are rather huge compared to the volume of the periarbuscular space. Fluxes between the compartments that strongly change the apoplastic concentration do not have a significant impact on the cytoplasmic concentrations; therefore, cytoplasmic concentrations were kept constant in the simulations. In the considered sector of the periarbuscular space, the concentrations could be contemplated without spatial imbalances as these gradients would quickly dissipate by diffusion in this tiny volume. Transport of sugar or phosphate across both membranes was mediated by H^+^/sugar (H/C) and H^+^/phosphate (H/P) cotransporters. H^+^-ATPases were gathered with other potential ionic conductances in a background conductance (Figure 1B).

### Mathematical description of transporter activities

**Figure 2:**
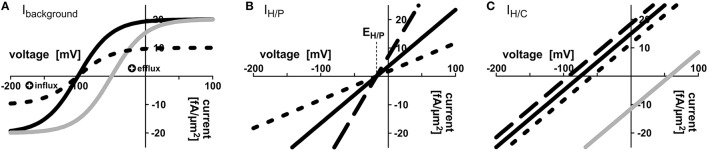
**Mathematical description of transporter activities. (A)** Voltage dependence of the background conductance I_background_ = I_BG_ = I_BGmax_× (1 − exp[−(V − V_0_) × F/(RT)])/(1 + exp[−(V − V_0_) × F/(RT)]); here V_0_ = −100 mV (*black curves*) or V_0_ = −50 mV (*gray curve*); I_BGmax_ = 20 fA/μ*m*^2^ (*solid lines*), I_BGmax_ = 10 fA/μ*m*^2^ (*dotted line*). **(B,C)** Voltage dependencies of the **(B)** H/P and **(C)** H/C cotransporters were approximated by first-order Taylor approximation. **(B)** I_H/P_ = G_H/P_× (V − RT/F × [2 × ln(H_apo_/H_cyt_) + ln(P_apo_/P_cyt_)]); G_H/P_ = 200 fA/(μ*m*^2^× V) (*solid line*), G_H/P_ = 400 fA/(μ*m*^2^× V) (*dashed line*), G_H/P_ = 100 fA/(μ*m*^2^× V) (*dotted line*). P_cyt_ = 2 mM, P_apo_ = 10 μM, pH_cyt_ = 7.0, pH_apo_ = 6.0, i.e. E_H/P_ = −17.3 mV. **(C)** I_H/C_ = 200 fA/(μ*m*^2^× V) × (V − RT/F × [ln(H_apo_/H_cyt_) + ln(C_apo_/C_cyt_)]); C_cyt_ = 2 mM (*solid lines*), C_cyt_ = 4 mM (*dashed line*), C_cyt_ = 1 mM (*dotted line*), C_apo_ = 10 μM (*black lines*) or C_apo_ = 2 mM (*gray line*). The basic settings of the transporter network were: ${\text{P}}_{\text{cyt}}^{\text{plant}}$ = 1.5 mM, ${\text{P}}_{\text{cyt}}^{\text{fungus}}$ = 3.0 mM, ${\text{C}}_{\text{cyt}}^{\text{plant}}$ = 2 mM, ${\text{C}}_{\text{cyt}}^{\text{fungus}}$ = 1 mM, pH_apo_ = 6.0, p${\text{H}}_{\text{cyt}}^{\text{plant}}$ = 7.0, p${\text{H}}_{\text{cyt}}^{\text{fungus}}$ = 7.0, ${\text{G}}_{\text{H}/\text{P}}^{\text{plant}}$ = 200 fA/(μ*m*^2^ × V), ${\text{G}}_{\text{H}/\text{P}}^{\text{fungus}}$ = 200 fA/(μ*m*^2^ × V), ${\text{G}}_{\text{H}/\text{C}}^{\text{plant}}$ = 200 fA/(μ*m*^2^ × V), ${\text{G}}_{\text{H}/\text{C}}^{\text{fungus}}$ = 200 fA/(μ*m*^2^ × V), ${\text{I}}_{\text{BGmax}}^{\text{plant}}$ = 20 fA/μ*m*^2^, ${\text{I}}_{\text{BGmax}}^{\text{fungus}}$ = 20 fA/μ*m*^2^, ${\text{V}}_{\text{p}}^{0}$ = ${\text{V}}_{0}^{\text{plant}}$ = −75 mV, ${\text{V}}_{\text{f}}^{0}$ = ${\text{V}}_{0}^{\text{fungus}}$ = −75 mV. The free-running parameters in *in silico* simulations were the apoplastic concentrations P_apo_ and C_apo_, and the voltages at the plant and fungal plasma membrane V_p_, V_f_.

Voltage dependence of the H^+^-ATPase driven background conductance was approximated by the sigmoidal function I_background_ = I_BG_ = I_BGmax_× (1 − exp[−(V_m_ − V_0_) × F/(RT)])/(1 + exp[−(V_m_ − V_0_) × F/(RT)]) (Figure 2A). Here, V_m_ is the voltage at the respective plasma membrane. The important feature of the H^+^-ATPase driven background current is that it reverts its direction at a certain voltage, V_0_, the equilibrium voltage. Positive of V_0_, I_BG_ is positive, which means that protons or other positive charges are exported from the cell, while negative of V_0_, I_BG_ is negative, which means that cations (e.g., H^+^, K^+^, ${\text{NH}}_{4}^{+}$) flow into the cell and/or anions (e.g., ${\text{NO}}_{3}^{-}$) flow out of the cell. The value V_0_ can be influenced by the activity of the H^+^-ATPase. A larger activity increases the efflux of positive charges and drives V_0_ to more negative voltages. I_BGmax_ is the maximal background current. RT/F ~ 25 mV is a factor composed of gas constant, temperature and Faraday constant. Also the current voltage characteristics of proton-coupled H^+^/X cotransporters I_H/X_ revert their direction at a certain voltage, E_H/X_, I_H/X_(E_H/X_) = 0. According to the enlarged Nernst-equation E_H/X_ depends on the concentrations of protons and the co-transported molecule X at both sides of the membrane: E_H/X_ = RT/F × [n_x_× ln(H_apo_/H_cyt_) + ln(X_apo_/X_cyt_)]/(n_x_+ z_x_) (Nour-Eldin et al., 2012). Here, z_x_ is the valence of the ion/metabolite X, z_C_ = 0 in case of sugar and z_P_ = −1 in case of phosphate, H_2_${\text{PO}}_{4}^{-}$; n_x_ is the number of protons transported per one particle X. Positive of E_H/X_, I_H/X_ is positive, which means that H^+^/X flow from the cell, while negative of E_H/X_ H^+^/X flow into the cell; I_H/X_ is negative. At voltages, which are not too far from this equilibrium voltage, the current voltage dependence of H^+^/X cotransporters can be approximated by a linear function according to the first-order Taylor approximation (Gajdanowicz et al., 2011) resulting in I_H/X_ = G_H/X_× (V_m_ − E_H/X_) (Figures 2B,C). Here, G_H/X_ is the conductance of the transporter. For E_H/C_ and E_H/P_, we set n_C_ = 1 without loss of generality for the sugar transporters (Carpaneto et al., 2005) and n_P_ = 2 for the phosphate transporters (Preuss et al., 2011). Different values, considering the conditions n_*C*_> 0 and n_*P*_> 1, respectively, do not change the results qualitatively. The transporter network is determined by the concentrations of H^+^, phosphate and carbon in the different compartments (${\text{H}}_{\text{cyt}}^{\text{plant}}$, H_apo_, ${\text{H}}_{\text{cyt}}^{\text{fungus}}$, ${\text{P}}_{\text{cyt}}^{\text{plant}}$, P_apo_, ${\text{P}}_{\text{cyt}}^{\text{fungus}}$, ${\text{C}}_{\text{cyt}}^{\text{plant}}$, C_apo_, ${\text{C}}_{\text{cyt}}^{\text{fungus}}$) and the 10-not fully independent-parameters ${\text{V}}_{\text{m}}^{\text{plant}}$, ${\text{V}}_{\text{m}}^{\text{fungus}}$, ${\text{I}}_{\text{BGmax}}^{\text{plant}}$, ${\text{I}}_{\text{BGmax}}^{\text{fungus}}$, ${\text{V}}_{0}^{\text{plant}}$ = ${\text{V}}_{\text{p}}^{0}$, ${\text{V}}_{0}^{\text{fungus}}$ = ${\text{V}}_{\text{f}}^{0}$, ${\text{G}}_{\text{H}/\text{P}}^{\text{plant}}$, ${\text{G}}_{\text{H}/\text{P}}^{\text{fungus}}$, ${\text{G}}_{\text{H}/\text{C}}^{\text{plant}}$, ${\text{G}}_{\text{H}/\text{C}}^{\text{fungus}}$. To reflect the pH buffer capacities, the pH-values in the apoplast and the cytosols were set constant to pH_apo_ = 6.0; p${\text{H}}_{\text{cyt}}^{\text{plant}}$ = p${\text{H}}_{\text{cyt}}^{\text{fungus}}$ = 7.0. Sugar-flux through SWEET channels was modeled as a diffusion process: J_SWEET_ = Diff_SWEET_ × (${\text{C}}_{\text{cyt}}^{\text{plant}}$ − C_apo_). The diffusion factor Diff_SWEET_ reflects the activity of SWEETs and was screened in the entire range from 0 to ∞.

### Computational cell biology

The behavior of the transporter network was mathematically simulated using Virtual Cell Modeling and Analysis Software (Figure 1B) developed by the National Resource for Cell Analysis and Modeling, University of Connecticut Health Center (Loew and Schaff, 2001). The model source code is provided in the Supplementary Material.

### Parameter screening and determination of marginal costs and marginal revenues

For each tested parameter the simulations were repeated with 28 logarithmically distributed values. As shown in the mathematical appendix (Supplementary Material), the dependency of the H/C- and H/P-fluxes on a certain parameter follows one of three possible equation types. The obtained H/P and H/C fluxes in equilibrium were used to determine the parameters in the respective equation. All non-linear fits were characterized by a regression coefficient of *r*^2^ = 1, i.e., no variance, indicating the precise description of the simulation results by the independently derived mathematical equations. The parameters were then used to calculate the first derivatives and thus the marginal costs (MC) and marginal revenues (MR). In our study, compared MC and MR are always expressed in different “currencies” (C per time or P per time). To be able to compare MR and MC, the MR-values were converted into the MC-currency by a factor that indicates the internal value of the traded good for the respective organism.

## Results

Previous wet-lab studies provided evidence that the fungus and the plant express proton-coupled phosphate and sugar transporters during AM formation (Harrison and van Buuren, 1995; Gianinazzi-Pearson, 1996; Harrison, 1996; Rausch et al., 2001; Harrison et al., 2002; Paszkowski et al., 2002; Javot et al., 2007; Helber et al., 2011; Casieri et al., 2013). In an attempt to test if these known transporters are sufficient to explain the nutrient exchange in AM symbiosis between plants and mycorrhizal fungi we took this transporter network as basis and also included H^+^-ATPase-driven background conductances in both membranes (Figure 1), then described all transporters mathematically (Figure 2) and carried out computational cell biology (dry-lab) experiments using VCell software (Loew and Schaff, 2001).

### A few molecules change largely the concentration in the tiny periarbuscular space

The interfacial apoplast between plant and fungus is a very tiny compartment with thickness of about 80–100 nm (Balestrini and Bonfante, 2005). If we now consider exemplarily a membrane patch of 1 × 1 μm, the volume between the plant and fungal plasma membrane patches is 0.8–1 × 10^−4^ pL. The transport of 500–600 sugar/phosphate molecules across the membrane patch changes the apoplastic sugar/phosphate concentration by 10 μM, a concentration change that can be achieved by the activity of a single transporter per patch within a second as proton-coupled transporters usually transport ~500 molecules per second (Derrer et al., 2013). These spatial conditions are considered in the cell biological simulations in order to reflect the real dimensions.

### The H^+^-coupled transporter network establishes a defined nutrient exchange system

**Figure 3:**
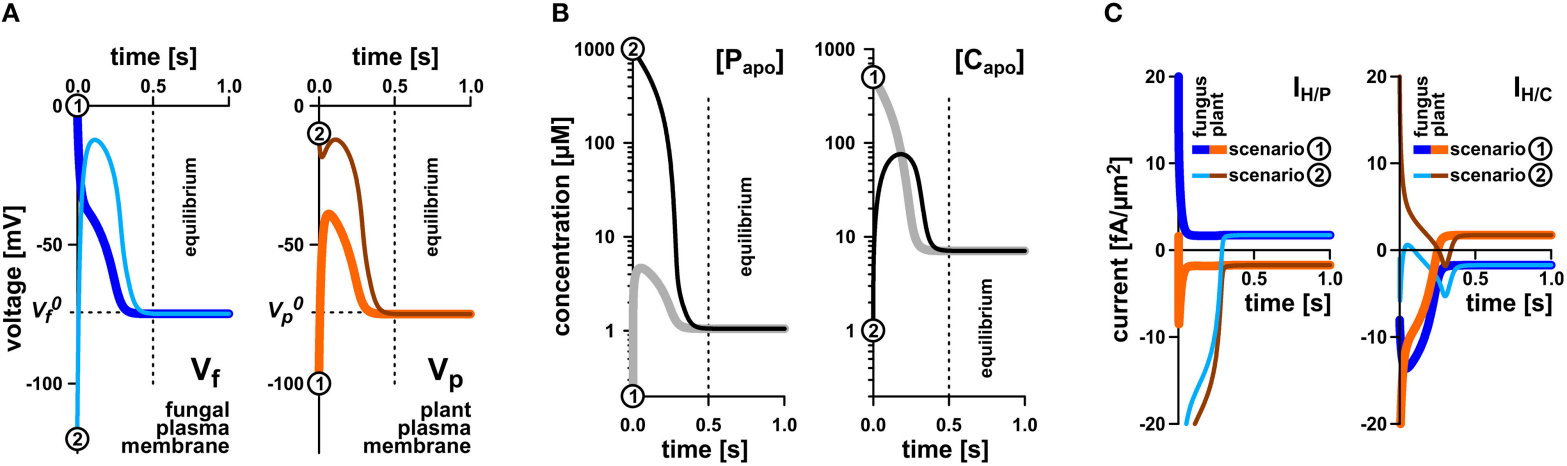
**Rapid equilibration of the nutrient uptake system. (A)** Time course of equilibration of the membrane voltages at the fungal plasma membrane (left) and the plant plasma membrane (right). (**B**) Time course of equilibration of the apoplastic phosphate (left) or sugar concentrations (right). (**C**) Time course of equilibration of proton-couplesd phosphate (I_H/P_, left) and sugar fluxes (I_H/C_, right) across the plant and fungal plasma membrane. Irrespective of the starting conditions (apoplastic P and C concentrations and voltages at the fungal and plant plasma membranes) the system reaches quickly an equilibrium that depends on the P and C concentrations in the cytosols of plant and fungus and the relative settings of the transporters. The equilibration processes for two different starting conditions [1, *thicker lines, gray* in **(B)**, *dark blue* or *orange* in **(A,C)**, and 2, *thinner lines, black* in **(B)**, *light blue* and *brown* in **(A,C)**] are shown. Please note that the equilibration time depends on the expression level of the transporters. In this study, a rather low expression level was chosen. The overall increase of the expression level would result in faster equilibration. In equilibrium there is still a continuous flux of phosphate from the fungus to the apoplast (**C**, left, positive current) and from the apoplast to the plant (**C**, left, negative current). Similarly, there is a constant flux of sugar from the plant via the apoplast to the fungus (**C**, right).

To elucidate whether the stability of the transporter network depends on the starting conditions, we tested a broad range of values. Irrespective of the starting condition the arbuscular transporter network (Figure 1) quickly attains a defined equilibrium state, at which the voltage at both membranes and the apoplastic sugar and phosphate concentrations are constant (Figures 3A,B). In Figure 3, the equilibration processes for two different starting conditions are shown. The apoplastic concentrations at equilibrium are in the micromolar or even sub-micromolar range (Figure 3B), which means that only a few phosphate or sugar molecules are left in the periarbuscular space. Interestingly, despite this emptiness, there is still a constant flux of phosphate from the fungus to the plant as indicated by a positive I_H/P_ current across the fungal membrane and a negative I_H/P_ current of the same amplitude across the plant membrane. This phosphate flux is accompanied by a sugar flux in the inverse direction (Figure 3C). Thus, the simple transporter network shown in Figure 1 appears to be not only involved in nutrient uptake but is also well suited to mediate the P/C-exchange observed in arbuscular fungal symbiosis. In this nutrient exchange, the H/C efflux at the plant is accompanied by a positive electric I_H/C_ current, which corresponds to a released electrochemical energy. This energy, in turn, is re-used by the plant for phosphate uptake by the electrogenic H/P-transporter. The positive I_H/C_ current compensates the negative I_H/P_ current. At the fungal membrane it is the other way round. Here, the released electrochemical energy from phosphate efflux energizes sugar uptake. The simple nutrient exchange system is highly energy-efficient.

### Demand and supply strategy in nutrient deal

**Figure 4:**
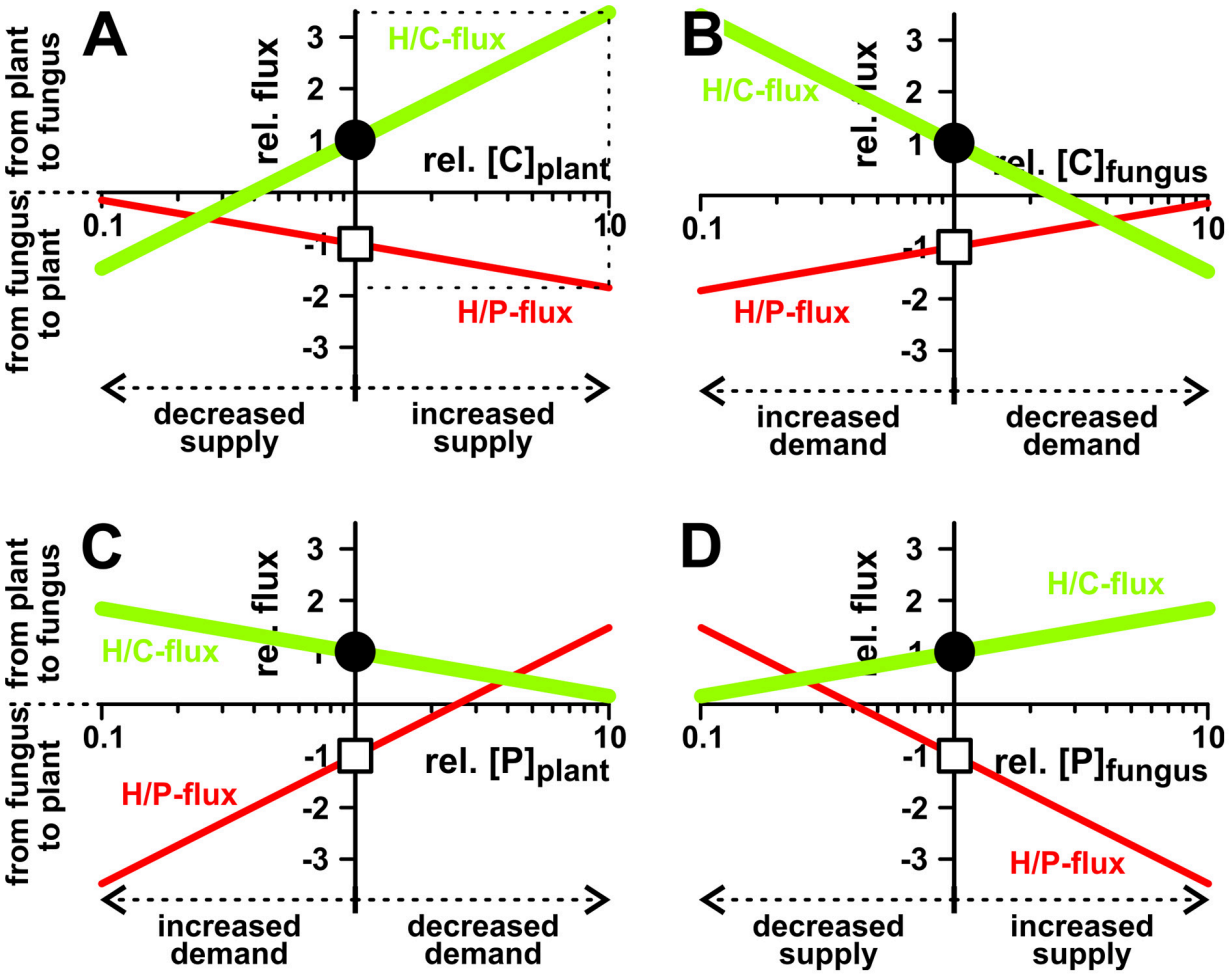
**Dependency of nutrient exchange on supply and demand**. Proton-coupled sugar (H/C, *green curves, lighter/bigger in gray scale*) and phosphate (H/P, *red curves, darker/smaller in gray scale*) fluxes in equilibrium as a function of varying cytosolic concentrations of **(A,B)** sugar ([C]) and **(C,D)** phosphate ([P]) in the plant **(A,C)** and the fungus **(B,D)**. For better comparison, concentrations, and fluxes were normalized. As reference we chose the H/C-flux across the plant membrane in equilibrium of the condition shown in Figure 3. The reference condition ([C]_plant_ = 2 mM, [C]_fungus_ = 1 mM, [P]_plant_ = 1.5 mM, [P]_fungus_ = 3 mM) is indicated by the position of the vertical axes and the circles (H/C) and squares (H/P). Concentration changes in [C] and [P] are displayed as relative changes. For instance, a value of [C]_plant_ = 4 mM correlates with rel.[C]_plant_ = 2, i.e., a doubling of the C-concentration in the plant, while halving the concentration to [C]_plant_ = 1 mM correlates with rel.[C]_plant_ = 0.5. Likewise, fluxes are displayed as relative fluxes: rel.flux = flux/(H/C-flux_in reference condition_). The dotted lines in **(A)** exemplify the reading of the figure: Compared to the reference condition, a 10-fold increase of [C]_plant_ (rel.[C]_plant_ = 10) would result in a 3.5-fold increase in H/C-flux (rel.flux = 3.5) and a 1.8-fold increase in H/P-flux (rel.flux = −1.8).

To get more insights into the dynamics of the transporter system, we systematically analyzed its properties in dry-lab experiments. At first, we repeated the simulations with different phosphate and sugar concentrations in the cytosol of the plant and the fungus (Figure 4). If the sugar concentration in the plant cytosol is increased, both H/P- and H/C-fluxes are stimulated (Figure 4A). Although one parameter is changed only, the increased C-gradient not only induces a larger sugar flux from the plant to the fungus but, due to the electrogenic nature of the H/C and H/P transport, also induces a larger phosphate flux in the inverse direction. The same phenomenon manifests if the phosphate concentration in the fungus is increased (Figure 4D), if the sugar concentration in the fungus is reduced (Figure 4B), or if the phosphate concentration in the plant is decreased (Figure 4C). Actually, the fluxes depend on the sugar and phosphate gradients between plant and fungus, which reflect “supply” and “demand” of the partners. More sugar in the plant cytosol (Figure 4A) or more phosphate in the fungal cytosol (Figure 4D) can be considered as a higher supply, while less sugar in the fungus (Figure 4B) or less phosphate in the plant (Figure 4C) represent a higher demand. Interestingly, the change of only one parameter is sufficient to enhance both, P and C transport. And in this it is irrelevant whether one partner provides higher supply (reciprocal reward (Kiers et al., 2011), Figures 4A,D) or gestures a higher demand (Figures 4B,C).

### Plant and fungus interact on equal terms

**Figure 5:**
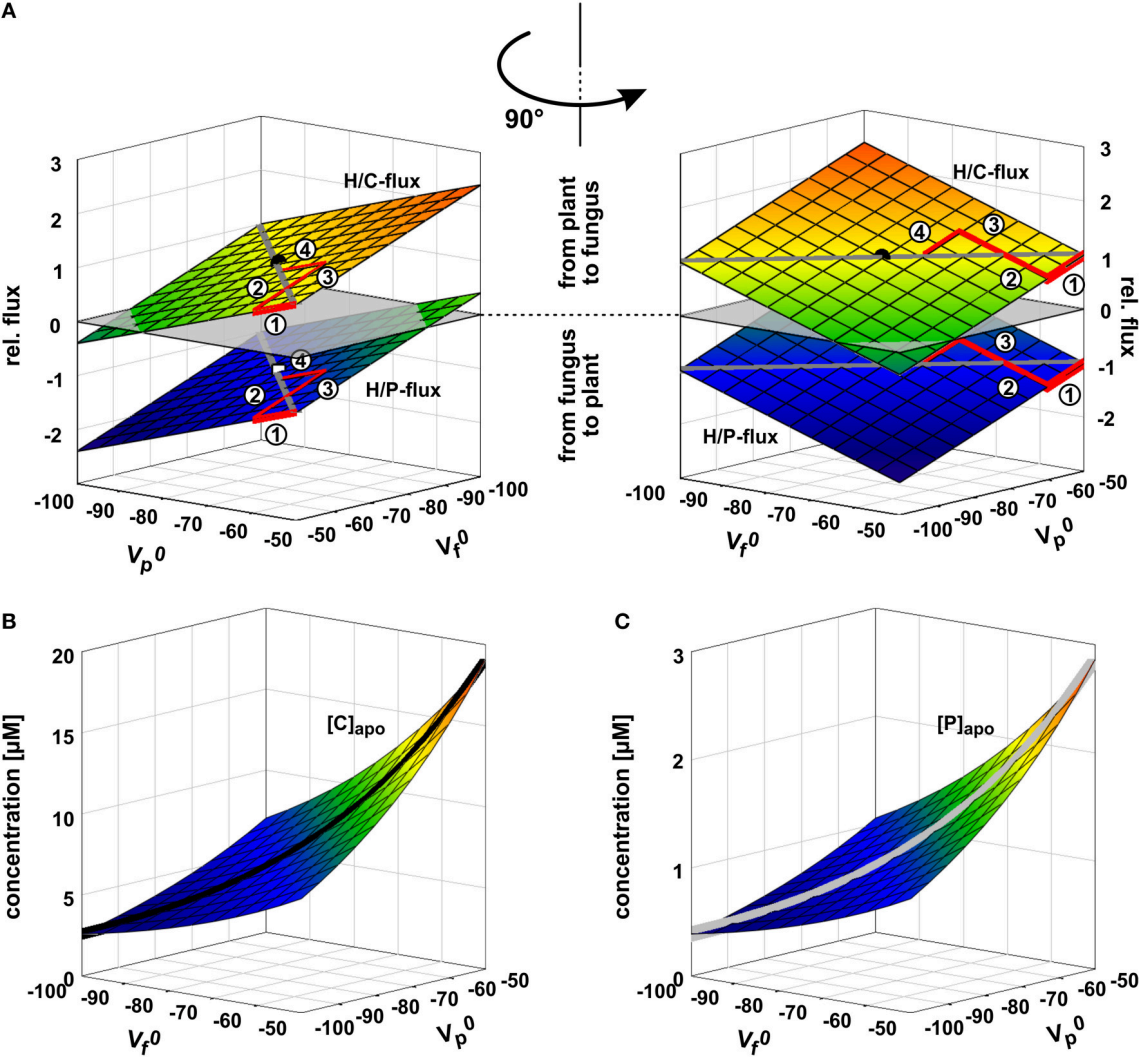
**Arms race in adjusting the zero-current voltages of the background conductance. (A)** Relative phosphate (H/P) and sugar (H/C) fluxes as a function of the zero-current voltages of the plant's (${\text{V}}_{\text{p}}^{0}$) and fungus' (${\text{V}}_{\text{f}}^{0}$) background conductances. ${\text{V}}_{\text{p}}^{0}$ and ${\text{V}}_{\text{f}}^{0}$ are strongly influenced by the activity of H^+^-ATPases; a higher pump activity drives the values to more negative voltages. If the plant unilaterally invests more pump energy, ${\text{V}}_{\text{p}}^{0}$ gets more negative and the plant benefits from a higher P-influx while reducing the C-efflux. In its extreme the plant could even achieve a C-flux from the fungus to the plant (negative values for H/C-flux). The gain of the plant is at the cost of the fungus. In return, the fungus can also invest pump energy and drive ${\text{V}}_{\text{f}}^{0}$ more negative with positive consequences for its C/P-balance but at the cost of the plant. The arms race between plant and fungus (1 → 2 → 3 → 4 → …) as equal partners ends in a draw at which ΔV_0_ = ${\text{V}}_{\text{f}}^{0}$ − ${\text{V}}_{\text{p}}^{0}$ = 0 with negative ${\text{V}}_{\text{p}}^{0}$ and ${\text{V}}_{\text{f}}^{0}$ values. The dot indicates the condition chosen for the other simulations in this study (${\text{V}}_{\text{p}}^{0}$ = ${\text{V}}_{\text{f}}^{0}$ = −75 mV). **(B,C)** Apoplastic carbon/sugar (**B**, [C]) and phosphate (**C**, [P]) concentrations at equilibrium of the transporter network as a function of ${\text{V}}_{\text{p}}^{0}$ and ${\text{V}}_{\text{f}}^{0}$. During the arms race between plant and fungus both concentrations decline (*black* and *gray curves*).

**Figure 6:**
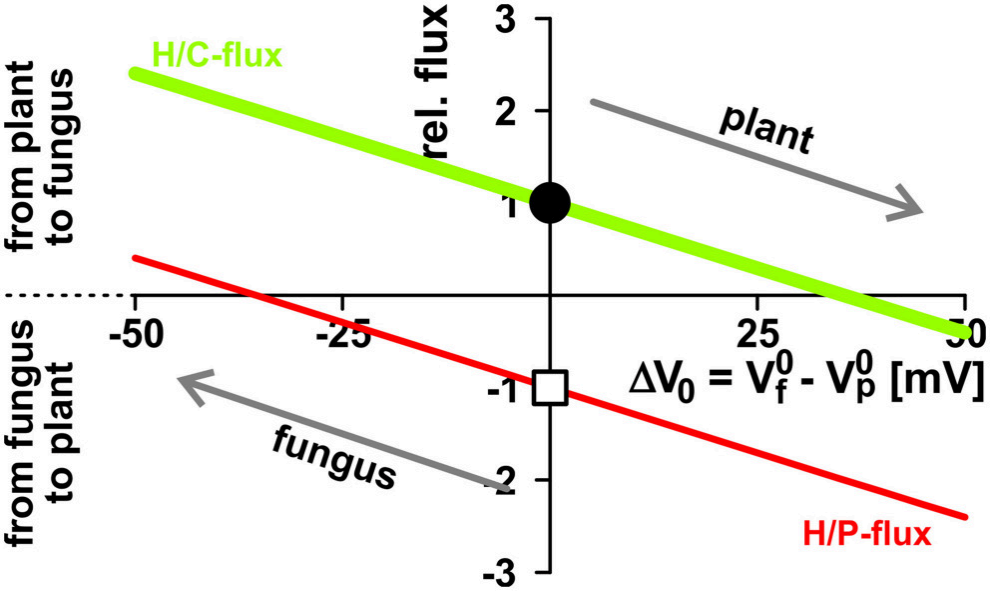
**Opposite interests of plant and fungus maintain a balance of power**. The activity of H^+^-ATPases influences the zero-current potentials (plant: ${\text{V}}_{\text{p}}^{0}$; fungus: ${\text{V}}_{\text{f}}^{0}$) of the background conductances at the plasma membranes. The proton-coupled sugar (H/C, *green, lighter/bigger in gray scale*) and phosphate (H/P, *red, darker/smaller in gray scale*) fluxes depend on the difference ΔV_0_ = ${\text{V}}_{\text{f}}^{0}$ − ${\text{V}}_{\text{p}}^{0}$. The plant has the interest to drive ${\text{V}}_{\text{p}}^{0}$ more negative (and therefore to drive ΔV_0_ more positive) to reduce the loss in sugar and to increase the gain in phosphate, while the fungus tends to reduce the loss of phosphate and to increase the gain of sugar by driving ${\text{V}}_{\text{f}}^{0}$ (and ΔV_0_) more negative. Between equal partners the different forces balance at ΔV_0_ = 0. For further details, see also Figure 5. For better comparison, fluxes were normalized, as explained in Figure 4, to the H/C-flux across the plant membrane in equilibrium of the condition shown in Figure 3 and displayed as relative changes. Circle (H/C) and square (H/P) indicate this reference condition.

**Figure 7:**
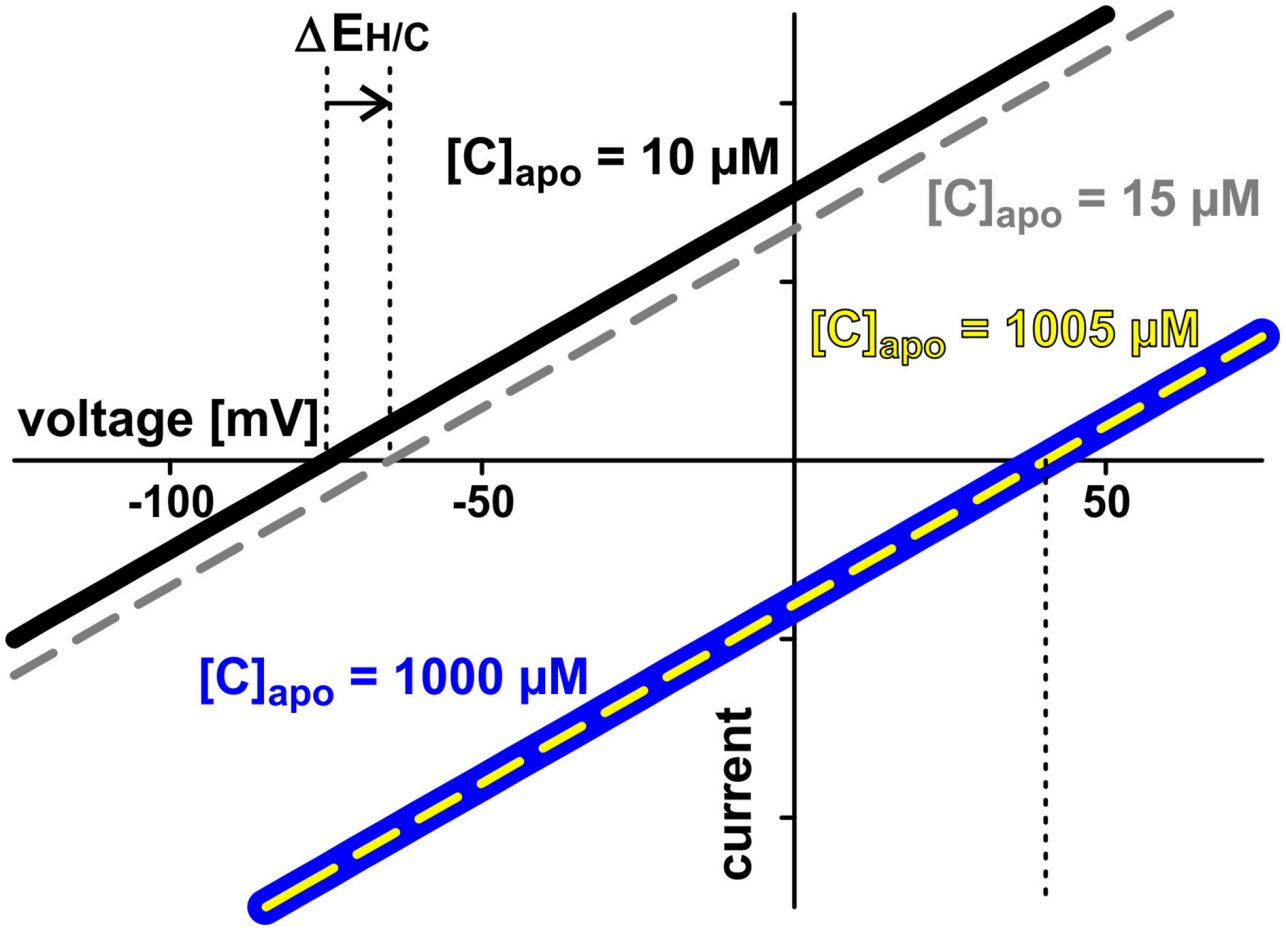
**Low apoplastic concentrations facilitate sensing by proton-coupled transporters**. Exemplarily the current voltage curves of a proton-coupled H/C-transporter are shown. The cytosolic sugar concentration is [C]_cyt_ = 2 mM in all cases. Two cases are considered: In case 1 (*black* and *gray curves*) the apoplastic concentration is initially [C]_apo_ = 10 μM (*black*). The increase by additional 5 μM shifts the current voltage characteristics to less negative voltages (ΔE_H/C_, *gray, dashed*). The transporter senses the 5 μM difference. The modified properties feedback on the entire transporter network. In case 2 (*blue* and *yellow curves*) the apoplastic concentration is initially [C]_apo_ = 1000 μM (*blue*) and increase of the same magnitude (5 μM) does not change the current voltage characteristics (*yellow*). The transporter cannot sense the difference between [C]_apo_ = 1000 μM and [C]_apo_ = 1005 μM. There is no feedback of the concentration change to the transporter network.

The two symbionts can control the nutrient fluxes not only via their cytosolic phosphate and sugar concentrations but also by regulating the activity of the transporters. If, for instance, the plant invests more energy to fuel H^+^-ATPases, its membrane voltage gets more negative (Figure 2A, shift from gray to black solid curve, ${\text{V}}_{\text{p}}^{0}$ gets more negative). As a consequence, the C-loss of the plant gets smaller while its P-gain gets larger (Figure 5A, 1). In comparison to the situation in Figure 3, now the plant energizes the P-uptake not only by the electrochemical energy of H/C-release, but invests energy from a different source (e.g., ATP hydrolysis). Such a situation is considered as standard if the plant is absorbing P from the surrounding soil. However, in AM symbiosis, the improvement of the plant's P/C-balance is at the cost of the fungus' C/P-balance. In return, the fungus can positively influence its C/P-balance by also investing more energy to fuel H^+^-ATPases resulting in a negative shift of ${\text{V}}_{\text{f}}^{0}$ and a more negative voltage at the fungal membrane (Figure 5A, 2). Remarkably, by larger energy investment the fungus could even invert the P-efflux into an influx; similarly, the plant could invert the C-efflux into an influx. Thus, as one organism can absorb both, C and P, each partner could in principle exploit the other. Plant and fungus are therefore in a kind of “arms race” with a “balance of power” at ${\text{V}}_{\text{p}}^{0}$ = ${\text{V}}_{\text{f}}^{0}$ (or ΔV_0_ = ${\text{V}}_{\text{f}}^{0}$ − ${\text{V}}_{\text{p}}^{0}$ = 0, Figures 5A, 6). As a consequence, apoplastic [P] and [C] are very low (Figures 5B,C; see also above). These low concentrations have a positive side-effect since it allows each partner to monitor the cooperativity of the other (Figure 7). In an example that should illustrate this fact we consider an H/C-transporter, a cytosolic C-concentration of [C]_cyt_ = 2 mM and a pH difference between cytosol and apoplast of 1. In case the apoplastic C-concentration is initially [C]_apo_ = 10 μM, the H/C-transporter does not transport in either direction if the membrane voltage is V = E_H/C_ ≈ −75 mV. More positive of E_H/C_ the H/C-flux is out of the cell while more negative of E_H/C_ it is into the cell (Figure 7, black curve). The increase of [C]_apo_ by additional 5 μM shifts this equilibrium voltage by about 10 mV more positive to E_H/C_ ≈ −65 mV (Figure 7, gray dashed curve). It can be observed that a change of [C]_apo_ by 5 μM converts an initial H/C-efflux into an influx, if the membrane voltage is kept at −70 mV. Now, considering the same change in concentration, in an upper range limit (from [C]_*apo*_ = 1000 μM to [C]_apo_ = 1005 μM), the equilibrium voltage is E_H/C_ ≈ + 40 mV (Figure 7, blue curve). The increase of [C]_apo_ by additional 5 μM has no remarkable effect neither on the equilibrium voltage, nor on the flux direction at a certain voltage (Figure 7, yellow dashed curve). Thus, due to the low apoplastic concentrations of the exchanged nutrients in the tiny volume of the periarbuscular space one partner can sense within seconds or even faster the activity and therefore cooperativity of the other symbiont.

**Figure 8:**
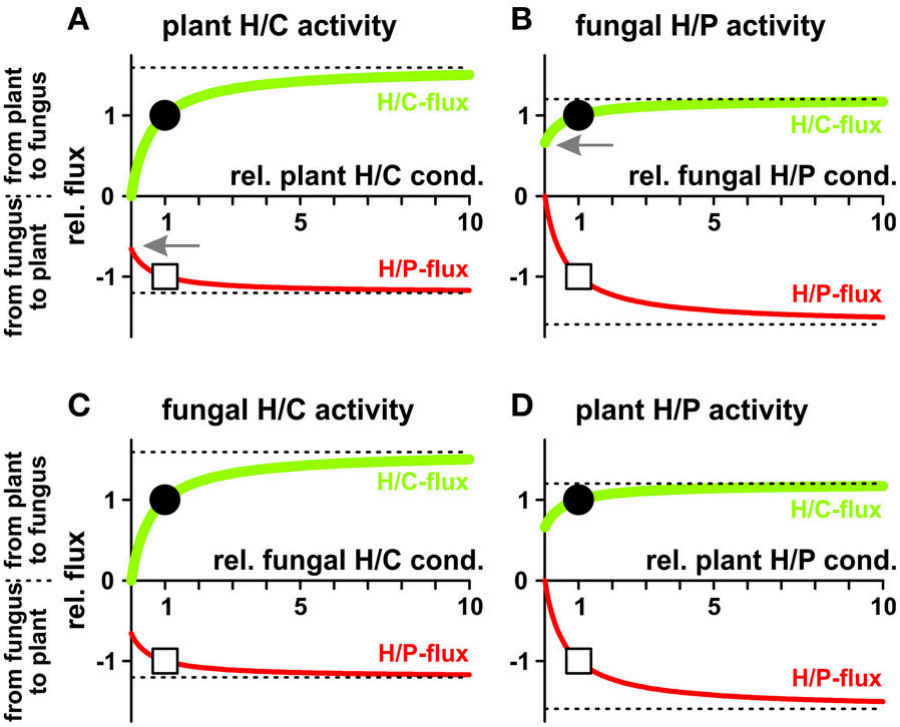
**Dependency of proton-coupled sugar (H/C, *green*) and phosphate (H/P, *red*) fluxes on the activity of plant and fungal transporters. (A)** Fluxes as a function of the activity of the plant H/C-transporter. **(B)** Fluxes as a function of the activity of the fungal H/P-transporter. **(C)** Fluxes as a function of the activity of the fungal H/C-transporter. **(D)** Fluxes as a function of the activity of the plant H/P-transporter. For better comparison, fluxes were normalized, as explained in Figure 4, to the H/C-flux across the plant membrane in equilibrium of the condition shown in Figure 3 and displayed as relative changes. Circle (H/C) and square (H/P) indicate the reference condition. If, *ceteris paribus*, all plant H/C-transporters are turned off, the sugar flux vanishes while there is still a significant phosphate flux from the fungus to the plant (**A**, arrow). If, *ceteris paribus*, all fungal H/P-transporters are shut down, the phosphate flux vanishes while there is still a significant sugar flux from the plant to the fungus (**B**, arrow). The dotted lines indicate the maximal fluxes that can be achieved at infinitely high expression and activity levels.

In a different scenario plant and fungus can regulate their proton coupled transporters to modify the fluxes for their benefits (Figure 8). Increasing the activity of any of the four transporter types increases the fluxes of both, sugar and phosphate. Nevertheless, if one partner may decide to reduce its nutrient loss, it does not need to sacrifice fully its nutrient gain. In principle, the plant could switch off the activity of its sugar transporters (H/C) and would still benefit from significant phosphate supply by the fungus (Figure 8A, arrow). In this case the plant acts solely with a proton pump-driven proton-coupled phosphate transporter and uses the energy from ATP hydrolysis for the uptake of phosphate. Similarly, the fungus could shut down its phosphate transporters without losing all sugar supply (H/P; Figure 8B, arrow) and would switch into a proton pump-driven uptake mode.

### Basic microeconomic principles provide the driving forces for a cooperative behavior

In their trade of nutrients plant and fungus are participants in a local economy. Therefore, we had to cross disciplines and analyzed the transporter system in scientific terms not only from a biological but also from an economical perspective. In the simplest notion both, plant and fungus, are considered as selfish partners in a bi-polar economy. They neither exhibit altruistic behavior nor any other long-term collaboration strategy. Instead, each is only interested in maximizing the gain for reasonable costs. The gain of the plant is accumulation of phosphate per time and that of the fungus is accumulation of sugar per time. The main cost for the plant is the loss of sugar per time and for the fungus is the loss of phosphate per time.

**Figure 9:**
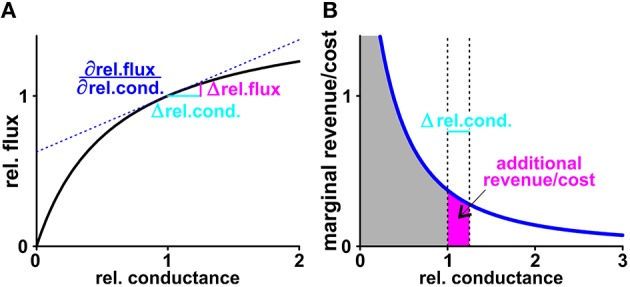
**Marginal revenue/marginal cost. (A)** The black curve shows exemplarily the dependency of the normalized sugar flux on the normalized conductance of the plant H/C-transporter. The normalization process results in unit-less values: Doubling the conductance from ${\text{G}}_{\text{H}/\text{C}}^{\text{plant}}$ = 200 fA/(μ*m*^2^×V) to 400 fA/(μ*m*^2^×V) is equivalent to a shift of the relative conductance from 1 to 2. This change increases the H/C-flux from 1.74 to 2.13 fA/μ*m*^2^, which is equivalent to an increase in the relative flux from 1 to 1.22. If the rel.conductance changes by the small amount Δrel.cond. (*light blue*) there is an additional H/C-flux (Δrel.flux, *magenta*) from the plant. At the limit △ rel.cond. → 0 the additional flux can be calculated as △ rel.flux = $\frac{\partial \text{rel}.\text{flux}}{\partial \text{rel}.\text{cond}.}\cdot$ △rel.cond. with the derivative of the black curve $\frac{\partial \text{rel}.\text{flux}}{\partial \text{rel}.\text{cond}.}$ at the starting point (*blue, dashed line*). **(B)** The derivative of the black curve is the marginal cost curve (*blue*). The surface below the curve down to the axis (*gray*) indicates the total cost. For a small change Δrel.cond. (*light blue*) the additional surface (*magenta*) indicates the additional cost superimposed by this change. Calculations of marginal revenues are equivalent to the considerations for “costs” outlined here in detail.

We started the economic considerations with a game theoretical scenario. When plant and fungus play the “game of nutrient exchange,” one partner may suddenly decide to cut off the other from nutrient supply, while still benefiting from an influx, albeit slightly reduced (Figure 8). A reasonable reaction of the cheated partner on such an event would be the cessation of supply for its part. Without activities of neither the H/C-transporter in the plant nor the H/P-transporter in the fungus there is no phosphate-sugar exchange anymore. Thus, at first glance, the system presented in Figure 1 does not appear to be well-suited for nutrient exchange as it would be stable in a Nash-equilibrium that is not favorable for both sides, similar to the well-known prisoners' dilemma. However, in contrast to the prisoners, who could choose between two options only, plant and fungus have a continuum of options and can adjust their transporter activities smoothly. In this case, the optimal condition, i.e., the Nash-equilibrium, can be determined by the relationship between marginal costs and marginal revenues (Figure 9). To assess which might be the optimal expression and activity level of a certain transporter, we compare the additional revenue for the respective organism with the additional costs when increasing the activity level a bit more. If the marginal revenue is still larger than the marginal costs it is worth for the organism to increase the activity level further until the marginal revenue equals the marginal costs. Upon a further increase of the transporter activity the marginal costs would be higher than the marginal revenue, which would not be optimal for the organism. Thus, the intersection of marginal revenue and marginal cost curves defines a stable economic equilibrium.

**Figure 10:**
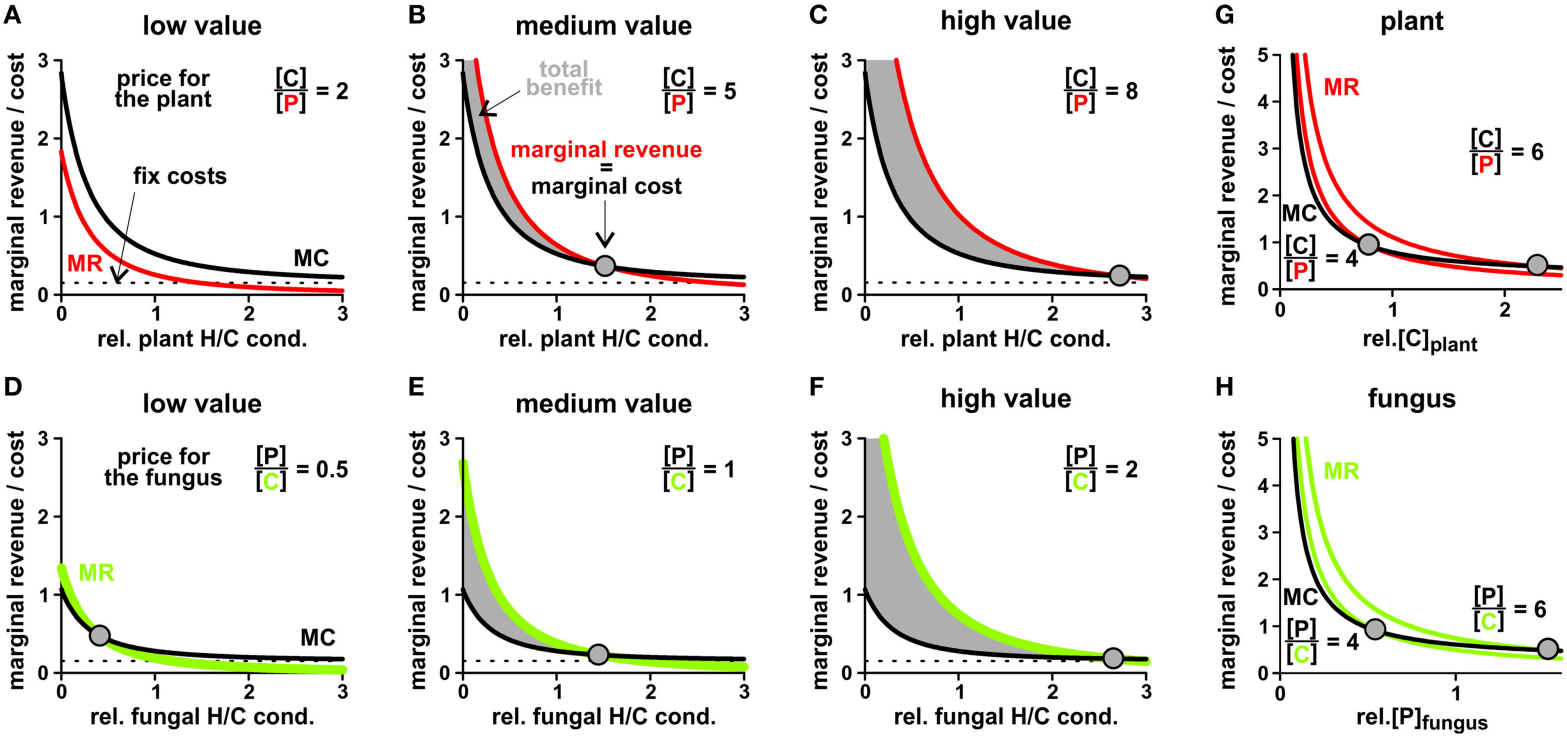
**Price-dependent economic optimisation of transporter activity and cytosolic concentrations**. **(A–C)** Adjustment of the plant H/C-transporter activity in three different scenarios: the phosphate provided by the fungus is **(A)** of low value (1 P values 2 C for the plant), **(B)** medium value (C/P = 5) and **(C)** high value for the plant (C/P = 8). In all cases, the revenue of the plant in terms of delivered P increases (Figure 8), however, the marginal revenue (*MR, red*) decreases with increasing transporter activity (relative plant H/C conductance). The plant “pays” a carbon source and may have additional fix costs for the expression and maintenance of functional transporters (marginal cost curve, *MC, black*). The intersection of the MR with the MC curve indicates the point of economic equilibrium, at which the marginal revenue equals the marginal costs (*dots*). At a higher H/C-transporter activity the additional costs of the plant would be higher than its additional revenue, while at a lower activity the plant would not make maximal profit. Please note that in **(A)** the two curves do not intersect; the economical equilibrium is at zero, i.e. no plant H/C-transporter activity. **(D–F)** Adjustment of the fungal H/C-transporter activity in three different scenarios: the carbon source provided by the plant is **(D)** of low value (2 C values 1 P for the fungus), **(E)** medium value (P/C = 1) and **(F)** high value for the fungus (P/C = 2). The marginal revenues for the carbon source (*MR, green*) are compared with the marginal cost curve (MC, *black*) for phosphate and potential fix costs. **(G,H)** Price-dependent economic optimisation of nutrient supply. **(G)** Marginal cost = additional carbon/sugar release (*MC, black*) and marginal revenue = additional phosphate gain (*MR, red*) as a function of the cytosolic sugar ([C]) concentration in the plant for two different plant-specific C/P values. With increasing C/P value the economic equilibrium shifts to higher cytosolic [C] levels. **(H)** Marginal cost = additional phosphate release (*MC, black*) and marginal revenue = additional carbon/sugar gain (*MR, green*) as a function of the cytosolic phosphate ([P]) concentration in the fungus for two different fungus-specific P/C values. With increasing P/C value the economic equilibrium shifts to higher cytosolic [P] levels. Please note: The MC and MR curves do not have units as they are the first derivatives of the unit-less relative P- and C-fluxes plus the fix costs in case of MC. To be able to compare MR with MC their different “currencies” (C per time or P per time) were converted by the indicated conversion factor, which indicates the individual value for the respective organism.

To evaluate the economically optimal activity of H/C-transporters in the plant plasma membrane, the marginal revenue is calculated from the H/P-flux curve in Figure 8A and the marginal cost from the H/C-flux curve. On top of these expenses other fixed costs for the expression and functional maintenance of the transporter can be considered. The plant generates a benefit as long as the marginal revenue curve is above the marginal cost curve (Figures 10A–C). This condition sets the economical limit for the activity of the H/C-transporters. As shown in Figure 10, the higher the plant values the traded phosphate the more H/C-transporters can be active. A low phosphate value can certainly result in the non-cooperative scenario described above, in which the plant does not maintain functional H/C-transporters (Figure 10A).

Based on economic principles we thus postulate the regulation of the plant H/C-transporter in response to the plant's phosphate status. Although this signaling cascade remains to be elucidated in wet-lab experiments, several experimental findings are well in line with such a mechanism. For instance, if the plant gets significant amounts of phosphate from other sources like mineral fertilizers or other symbiotic organisms, the phosphate provided by the fungus is of lesser value for the plant and the plant declines its cooperativity toward the fungus (Cowden and Peterson, 2009; Verbruggen and Kiers, 2010; Kiers et al., 2011; Wyatt et al., 2016).

At the fungal side of the periarbuscular space we can consider the microeconomic situation for the activity of the fungal H/C-transporter. In this case, sugar is the desired good and phosphate is the currency to pay with. As shown in Figures 10D–F, from an economic point of view it makes sense for the fungus to maintain a high H/C-transporter activity even for a lower carbon value.

The economically optimal activity of phosphate transporters can be analyzed in a similar manner with analogous results: In economically optimal conditions the fungal H/P-transporter activity strongly depends on the value that sugar has for the fungus while throttling the plant H/P-transporter is not as price dependent (not shown).

Also the sugar and phosphate supply is subject to market forces (Figures 4, 10G,H). Although one partner could theoretically achieve nutrient gain for zero or even negative costs by keeping the cytosolic concentrations very low, the total benefit is maximized if significant amounts of P and C, respectively, are provided. Also here, the optimal conditions depend on the organism-specific internal value of the traded good (Figures 10G,H). Thus, the simple transporter system is highly flexible and both partners can control nutrient exchange at several set screws.

### Increasing complexity by expanding the model

**Figure 11:**
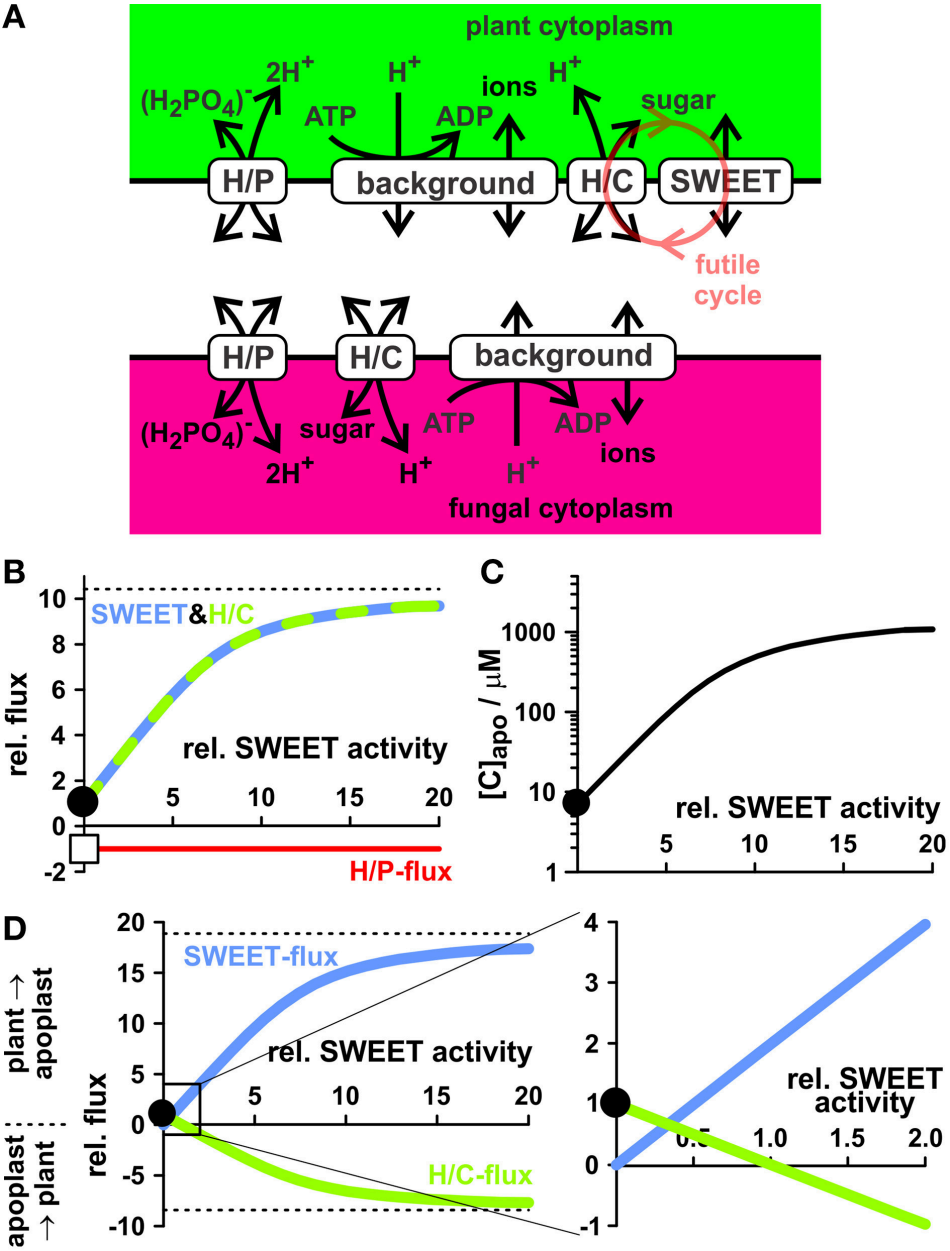
**The futile cycling of sugar. (A)** Transporter network that allows the efflux of electroneutral sugar molecules from the plant to the periarbuscular space via proton-coupled transporters (H/C) and sugar channels (SWEET). The red circle indicates the futile sugar cycle that results from the co-existence of active H/C and SWEET transporters. **(B)** Dependency of the sugar/phosphate exchange between plant and fungus on the activity of SWEET channels. Without active SWEET channels, there is a constant sugar flux from the plant cytosol to the periarbuscular space and from the periarbuscular space to the fungus. All displayed fluxes are normalized to this H/C-flux (*black circle*). In return there is a constant flux of phosphate from the fungus via the periarbuscular space to the plant (*white square*). Upon increasing the activity of the SWEET channel, this phosphate flux is not affected while the sugar flux from the plant to the fungus rises. The dotted line indicates the maximal flux that can be achieved at infinitely high SWEET activity levels. **(C)** Activation of SWEET channels floods the apoplast with sugar. Equilibrium apoplastic sugar concentration as a function of the activity of SWEET channels. **(D)** Sugar flux through H/C and SWEET from the plant to the apoplast and from the apoplast to the plant as a function of the activity of SWEET channels. The right panel is an enlargement of the black rectangle in the left panel. Without active SWEET channels the transmembrane sugar gradient drives H^+^/sugar efflux via H/C-transporters. Here, each sugar molecule transports a proton from the plant cytosol to the apoplast and assists the H^+^-ATPase in charging the proton gradient that can be harvested by the H/P transporter for phosphate uptake. With increasing SWEET activity more and more sugar flows without piggybacked proton from the cytosol to the apoplast and is re-taken up by H/C-transporters. Activity of SWEET channels was normalized, so that at a relative activity of 1 the flux through H/C-transporters reverts its direction.

The presented minimal model for a nutrient trade system explains very well observed phenomena. It can thus serve as benchmark to evaluate the performance of alternative and/or enlarged models for nutrient exchange. P and C sources might be transported not only via proton-coupled transporters but by diffusion facilitators. For instance, P could also be transported via phosphate-permeable anion channels (Dreyer et al., 2012) and C could be transported in neutral form as sugar through sugar channels (Chen et al., 2010) or as organic acid through channels of the ALMT type (Dreyer et al., 2012). Models that also contain ion channels are predominantly developed for guard cells (Hills et al., 2012; Blatt et al., 2014; Minguet-Parramona et al., 2016). Here, we increased the complexity of the minimal model by integrating sugar channels of the SWEET type for sugar release (Figure 11) from the plant as they have been found to be also upregulated during arbuscule formation (Manck-Götzenberger and Requena, 2016). However, under the dry lab conditions tested in this work, the functional expression of sugar diffusion facilitators (SWEET) in addition to the H/C-transporter impairs the plant's nutrient deal. Without active SWEET channels, there is a constant sugar flux from the plant cytosol to the periarbuscular space and from the periarbuscular space to the fungus (Figure 11B, black circle). In return there is a constant flux of phosphate from the fungus via the periarbuscular space to the plant (Figure 11B, white square). Upon increasing the activity of SWEET channels, this phosphate flux is not affected while the sugar flux from the plant to the fungus rises. Jointly, also the apoplastic sugar concentration rises (Figure 11C) and the altered H^+^/sugar gradient modifies the transport direction of the H/C-transporters. They turn from efflux transporters into influx transporters (Figure 11D) and are employed to retrieve sugar molecules from the periarbuscular space using the electrochemical proton gradient established by H^+^-ATPases. Thus, the co-existence of active proton-coupled H/C-transporters and C diffusion facilitators transporting the same carbon source would not only promote the dissipation of the energy of the sugar gradient, it would also increase the apoplastic C concentration, with the concomitant risk of losing carbon from the periarbuscular space by diffusion, and would additionally provoke a waste of valuable energy via the creation of a futile cycle of sugar efflux and retrieval. In order to avoid this futile cycle, the model suggests that either the SWEETs are tightly regulated or separated from regions where proton-coupled co-transporters exist, which use the same substrate as the SWEETs.

## Discussion

In this study we elaborated the thermodynamic flexibility of the simple transporter system shown in Figure 1 using computational cell biology. This approach allowed us to gain insights into the system that are far beyond the reach of any wet-lab experimental technique available at the moment. To evaluate the robustness of the obtained results we assessed the foundations and the limits of the model.

### The model is based on wet-lab experimental evidence

Extensive molecular biology work and proteomic approaches provide clear evidence that proton-coupled phosphate and sugar transporters are present in the periarbuscular zone (Harrison and van Buuren, 1995; Gianinazzi-Pearson, 1996; Harrison, 1996, 2012; Rausch et al., 2001; Harrison et al., 2002; Paszkowski et al., 2002; Karandashov and Bucher, 2005; Javot et al., 2007; Helber et al., 2011; Doidy et al., 2012). However, at the moment it is not clear whether also other types of transporters like sugar channels or phosphate-permeable anion channels may contribute to the nutrient exchange as well. We therefore consider the model shown in Figure 1 as a basic model that can be extended in future computational cell biology studies (as exemplarily shown in Figure 11) in order to evaluate the thermodynamic advantages and disadvantages of the expression and activation of these newly discovered transporters. A subsequent justification of the model design is provided by the conclusions drawn from this study. The results explain the big advantages of handling the nutrient exchange via H^+^-coupled transporters, as in this case each organism maintains tight control over the exchange process (Figure 7).

### The simulation covers a broad range of biological realities

The model is characterized by a large set of 20 free or partially dependent parameters. Namely these are: the distance between the two membranes, the concentrations of protons, sugar and phosphate in the three compartments, the activity/expression levels of the six transporters, the equilibrium voltages ${\text{V}}_{\text{p}}^{0}$ and ${\text{V}}_{\text{f}}^{0}$ of the H^+^-ATPase-dependent background conductances, and the voltages at the two membranes. To gain confidence in the reliability of the simulations the parameters were carefully assessed: (i) The size of the periarbuscular space was set to 100 nm, a value reported in literature (Balestrini and Bonfante, 2005). This value was used to calculate the volume of the apoplast between both membranes and to determine the concentration changes during the simulations. The increase or decrease of this value in the simulation by a factor of 10 (1 μm and 10 nm, respectively) affected only the initial equilibration process (time interval before the dashed line in Figure 3) by factor ~3 and ~0.8, respectively, but left the equilibrium conditions unaffected. Because in this study we considered the system always in its equilibrium, the results presented here do not depend on the exact size of the interfacial apoplast between plant and fungus. (ii) The pH values were set to physiological pH7.0 in the cytosols and to pH6.0 in the apoplast. A different apoplastic pH in the range < pH7.0 does not affect qualitatively the results. A far lower pH (Guttenberger, 2000) would result in even lower nutrient concentrations in the periarbuscular space. (iii) The activity levels of the six transporters were normalized to the activity of the plant H/C-transporter in a reference condition (Figure 3). When we changed all activities in the reference condition by factor 0.1 or factor 10 only the initial equilibration process was affected. It slowed down or increased by a factor of 10. Thus, the behavior of the system in equilibrium does not depend on the absolute expression/activity level of the transporters, but only on the relative activity levels toward each other. (iv) The other parameters were either determined in the simulations ([C_apo_], [P_apo_], V_p_, V_f_) or they were screened in the entire reasonable interval (from −∞ to ∞ for ${\text{V}}_{\text{p}}^{0}$ and ${\text{V}}_{\text{f}}^{0}$, or from 0 to ∞ for the relative transporter activities and for [C]_plant_, [C]_fungus_, [P]_plant_, [P]_fungus_). In summary we can state that we have explored the entire parameter space of the system. Therefore, the results presented here represent a broad range of biological realities, rather than being restricted to a particular set of parameters.

### The molecular model explains macroscopic observations

The transporter network shown in Figure 1 reflects the basic transporters needed by the plant and the fungus to selfishly accumulate phosphate and sugar from the closer environment. It is widely assumed that the bidirectional mutualism observed in arbuscular mycorrhizal symbiosis must involve other, so far unknown, transporters that mediate the efflux of phosphate from the fungus and of carbohydrates from the plant (Bonfante and Genre, 2010; Smith and Smith, 2011; Johri et al., 2015). Although we do not exclude the involvement of other transporters, here we provide evidence by computational cell biology that the simple system of proton-coupled transporters would be sufficient for the exchange of phosphate and sugar between plant and fungus at the arbuscular interface. In this special environment the proton-coupled H/P- and H/C-transporters ensure very low apoplastic sugar and phosphate concentrations. Under these conditions, the Nernst-equilibrium of the proton-coupled transporters is at moderately negative, physiological voltages and slight variations in membrane voltage and/or concentrations can change the flux direction through the transporters. Thus, H/P- and H/C-transporters can function as both, uptake and release pathways; the direction of flux depends on the electrochemical gradients. If plant and fungus optimize their economic benefits everyone for themselves, they establish a robust, tightly controllable, long-lasting trade in phosphate and sugar. The properties of the plant fungal P/C-economics of the transporter network (Figure 1) are well in line with those observed in ecological wet-lab experiments (Kiers et al., 2011). In other words, our model predicts precisely macroscopic observations. Indubitably, the dry-lab experiments presented here cannot provide an airtight proof of the accuracy of the working model but, the evidence provided manifests its validity.

### Competition—the basis of cooperation in AM symbiosis?

It might shake our idealistic picture of mutualist symbiosis when stating that plant and fungus compete with each other for the same resources. However, the model presented in this study paradoxically explains the nutrient exchange between plant and fungus with the simple assumption that each organism is only interested in maximizing the gain for reasonable costs. Such self-organizing processes are well-known in economics and were first described in 1776 by the Scottish philosopher Adam Smith (1776) for a selfish human economy. Adapted to AM symbiosis the famous statement of Smith would read: *both actors, plant and fungus, intending only their own gains are led by an invisible hand to promote an end that was no part of their intentions*.

## Author contributions

ID conceived the project and supervised the research. SS, BV, DB, JG, TS, and ID planned and designed computational cell biology experiments; and analyzed the data. JG, TS, and ID wrote the manuscript. All authors had intellectual input on the project and commented on the manuscript.

## Funding

This work was supported by the interdisciplinary Ph.D. program of the Universidad de Talca (SS, BV, DB) and the FONDECYT grant N° 1150054 of the Comisión Nacional Científica y Tecnológica of Chile (ID, JG, TS).

### Conflict of interest statement

The authors declare that the research was conducted in the absence of any commercial or financial relationships that could be construed as a potential conflict of interest.
