# Supplementary material for: Cooperation through Competition—Dynamics and Microeconomics of a Minimal Nutrient Trade System in Arbuscular Mycorrhizal Symbiosis
Source: Front Plant Sci. 2016 Jun 27;7:912. doi: 10.3389/fpls.2016.00912 (PMC4921476; doi:10.3389/fpls.2016.00912)
Supplement: Supplementary file 3 [file DataSheet3.pdf]

Mathematical Appendix - Analyses of the transporter network

To understand the thermodynamic flexibility of the transporter network in the periarbuscular interface in general, the system was analyzed in mathematical terms. As outlined in the Material and Methods section, the current-voltage characteristics of the proton-coupled transporters were approximated as linear functions of the membrane voltage by first-order Taylor approximation and described as:

$$I_{H/C-plant} = A \cdot \left(V_p - \frac{RT}{F} \cdot ln \left(\frac{[H_{apo}]}{[H_{plant-cyt}][C_{plant-cyt}]} \right) - \frac{RT}{F} \cdot ln([C_{apo}]) \right) = A \cdot (V_p - V_p^C - V_{apo}^C) \quad \text{(eq. 01)}$$

$$I_{H/P-plant} = B \cdot \left(V_p - \frac{RT}{F} \cdot ln \left(\frac{[H_{apo}]^2}{[H_{plant-cyt}]^2[P_{plant-cyt}]} \right) - \frac{RT}{F} \cdot ln([P_{apo}]) \right) = B \cdot (V_p - V_p^P - V_{apo}^P) \quad \text{(eq. 02)}$$

$$I_{H/C-fungus} = D \cdot \left(V_f - \frac{RT}{F} \cdot ln \left(\frac{[H_{apo}]}{[H_{fungus-cyt}][C_{fungus-cyt}]} \right) - \frac{RT}{F} \cdot ln([C_{apo}]) \right) = D \cdot (V_f - V_f^C - V_{apo}^C) \quad \text{(eq. 03)}$$

$$I_{H/P-fungus} = E \cdot \left(V_f - \frac{RT}{F} \cdot ln \left(\frac{[H_{apo}]^2}{[H_{fungus-cyt}]^2[P_{fungus-cyt}]} \right) - \frac{RT}{F} \cdot ln([P_{apo}]) \right) = E \cdot (V_f - V_f^P - V_{apo}^P) \quad \text{(eq. 04)}$$

with

$$V_p^C = \frac{RT}{F} \cdot ln \left(\frac{[H_{apo}]}{[H_{pplant-cyt}][C_{plant-cyt}]} \right) \quad \text{(eq. 05)}$$

$$V_{apo}^C = \frac{RT}{F} \cdot ln([C_{apo}]) \quad \text{(eq. 06)}$$

$$V_p^P = \frac{RT}{F} \cdot ln \left(\frac{[H_{apo}]^2}{[H_{plant-cyt}]^2[P_{plant-cyt}]} \right) \quad \text{(eq. 07)}$$

$$V_{apo}^P = \frac{RT}{F} \cdot ln([P_{apo}]) \quad \text{(eq. 08)}$$

$$V_f^C = \frac{RT}{F} \cdot ln \left(\frac{[H_{apo}]}{[H_{fungus-cyt}][C_{fungus-cyt}]} \right) \quad \text{(eq. 09)}$$

$$V_f^P = \frac{RT}{F} \cdot ln \left(\frac{[H_{apo}]^2}{[H_{fungus-cyt}]^2[P_{fungus-cyt}]} \right) \quad \text{(eq. 10)}$$

A (=$G_{H/C}^{plant}$), *B* (=$G_{H/P}^{plant}$), *D* (=$G_{H/C}^{fungus}$), and E (=$G_{H/P}^{fungus}$) denote the conductances of the plant H/C-, the plant H/P-, the fungal H/C-, and the fungal H/P-transporter, respectively. V_p is the voltage at the plant plasma membrane and V_f the voltage at the fungal plasma membrane.

The background current voltage characteristics were described by sigmoidal curves as

$$I_{BG-plant} = I_{BGmax-plant} \cdot \frac{1 - e^{-\left(\frac{F}{RT} \cdot [V_p - V_p^0]\right)}}{1 + e^{-\left(\frac{F}{RT} \cdot [V_p - V_p^0]\right)}} \quad \text{(eq. 11)}$$

$$I_{BG-fungus} = I_{BGmax-fungus} \cdot \frac{1 - e^{-\left(\frac{F}{RT} \cdot [V_f - V_f^0]\right)}}{1 + e^{-\left(\frac{F}{RT} \cdot [V_f - V_f^0]\right)}} \quad \text{(eq. 12)}$$

For better mathematical handling the functions were linearized in the vicinity of V_p^0 and V_f^0 by first order Taylor-approximation:

$$I_{BG-plant} \approx I_{BGmax-plant} \cdot \frac{1}{2} \cdot \frac{F}{RT} \cdot (V_p - V_p^0) = G \cdot (V_p - V_p^0) \quad \text{(eq. 13)}$$

$$I_{BG-fungus} \approx I_{BGmax-fungus} \cdot \frac{1}{2} \cdot \frac{F}{RT} \cdot (V_f - V_f^0) = K \cdot (V_f - V_f^0) \quad \text{(eq. 14)}$$

These functions describe linear curves that intersect the voltage axis at V_p^0 and V_f^0, respectively, and have the same slope as the sigmoidal I_{BG} curves at the respective intersection points.

Thermodynamic equilibrium

In equilibrium the membrane voltages V_p and V_f, as well as the concentrations $[P_{apo}]$ and $[C_{apo}]$ are constant. This implies that the following four conditions are fulfilled:

(i) the flux of sugar across the fugal plasma membrane compensates the flux of sugar across the plant plasma membrane; otherwise sugar would accumulate or dissipate in the apoplast and $[C_{apo}]$ would not be constant

$$I_{H/C-plant} + I_{H/C-fungus} = 0 \qquad \text{(eq. 15)}$$

(ii) the flux of phosphate across the fugal plasma membrane compensates the flux of phosphate across the plant plasma membrane; otherwise phosphate would accumulate or dissipate in the apoplast and $[P_{apo}]$ would not be constant

$$I_{H/P-plant} + I_{H/P-fungus} = 0 \qquad \text{(eq. 16)}$$

(iii) the charge net flux across the plant plasma membrane is zero; otherwise the net charge flux would change the membrane voltage V_p

$$I_{H/C-plant} + I_{H/P-plant} + I_{BG-plant} = 0 \qquad \text{(eq. 17)}$$

(iv) the charge net flux across the fungal plasma membrane is zero; otherwise the net change flux would change the membrane voltage V_f

$$I_{H/C-fungus} + I_{H/P-fungus} + I_{BG-fungus} = 0 \qquad \text{(eq. 18)}$$

By replacing the parameters in equations (15-18) with those of equations (01-04) and (13-14), the equilibrium condition can be described by the following linear equation system:

$$\begin{cases} (i) \quad 0 = A \cdot \left(V_p - V_p^C - V_{apo}^C\right) + D \cdot \left(V_f - V_f^C - V_{apo}^C\right) \\ (ii) \quad 0 = B \cdot \left(V_p - V_p^P - V_{apo}^P\right) + E \cdot \left(V_f - V_f^P - V_{apo}^P\right) \\ (iii) \quad 0 = A \cdot \left(V_p - V_p^C - V_{apo}^C\right) + B \cdot \left(V_p - V_p^P - V_{apo}^P\right) + G \cdot \left(V_p - V_p^0\right) \\ (iv) \quad 0 = D \cdot \left(V_f - V_f^C - V_{apo}^C\right) + E \cdot \left(V_f - V_f^P - V_{apo}^P\right) + K \cdot \left(V_f - V_f^0\right) \end{cases} \qquad \text{(eq. 19)}$$

or in matrix form:

$$\begin{pmatrix} A & D & -(A+D) & 0 \\ B & E & 0 & -(B+E) \\ (A+B+G) & 0 & -A & -B \\ 0 & (D+E+K) & -D & -E \end{pmatrix} \times \begin{pmatrix} V_p \\ V_f \\ V_{apo}^C \\ V_{apo}^P \end{pmatrix} = \begin{pmatrix} A \cdot V_p^C + D \cdot V_f^C \\ B \cdot V_p^P + E \cdot V_f^P \\ A \cdot V_p^C + B \cdot V_p^P + G \cdot V_p^0 \\ D \cdot V_f^C + E \cdot V_f^P + K \cdot V_f^0 \end{pmatrix}$$

This system has exactly one solution for the 4 values V_p, V_f, V_{apo}^C, and V_{apo}^P that depends on the activity levels of the transporters (*A*, *B*, *D*, *E*, *G*, *K*), the concentrations of P and C in the plant and the fungus (V_p^C, V_f^C, V_p^P, V_f^P) and the zero values of the background conductances (V_p^0, V_f^0). This solution can be used to calculate the currents through the different transporters in equilibrium yielding

$$I_{H/C}^{trade} = I_{H/C-plant}^{equilibrium} = -I_{H/C-fungus}^{equilibrium} = \frac{AD}{A+D} \cdot \frac{\frac{GK}{G+K}\cdot\left(V_{pf}^{C}-\Delta V_0\right)+\frac{BE}{B+E}\cdot\left(V_{pf}^{C}+V_{fp}^{P}\right)}{\frac{AD}{A+D}+\frac{BE}{B+E}+\frac{GK}{G+K}} \quad \text{(eq. 20)}$$

$$I_{H/P}^{trade} = I_{H/P-fungus}^{equilibrium} = -I_{H/P-plant}^{equilibrium} = \frac{BE}{B+E} \cdot \frac{\frac{GK}{G+K}\cdot\left(\Delta V_0+V_{fp}^{P}\right)+\frac{AD}{A+D}\cdot\left(V_{pf}^{C}+V_{fp}^{P}\right)}{\frac{AD}{A+D}+\frac{BE}{B+E}+\frac{GK}{G+K}} \quad \text{(eq. 21)}$$

$$I_{BG-plant}^{equilibrium} = -I_{BG-fungus}^{equilibrium} = \frac{GK}{G+K} \cdot \frac{\frac{AD}{A+D}\cdot\left(\Delta V_0-V_{pf}^{C}\right)+\frac{BE}{B+E}\cdot\left(\Delta V_0+V_{fp}^{P}\right)}{\frac{AD}{A+D}+\frac{BE}{B+E}+\frac{GK}{G+K}} \quad \text{(eq. 22)}$$

with $$V_{pf}^{C} = V_f^C - V_p^C = \frac{RT}{F}\cdot ln\left(\frac{[H_{plant-cyt}][C_{plant-cyt}]}{[H_{fungus-cyt}][C_{fungus-cyt}]}\right) \approx \frac{RT}{F}\cdot ln\left(\frac{[C_{plant-cyt}]}{[C_{fungus-cyt}]}\right) \quad \text{(eq. 23)}$$

$$V_{fp}^{P} = V_p^P - V_f^P = \frac{RT}{F}\cdot ln\left(\frac{[H_{fungus-cyt}]^2[P_{fungus-cyt}]}{[H_{plant-cyt}]^2[P_{plant-cyt}]}\right) \approx \frac{RT}{F}\cdot ln\left(\frac{[P_{fungus-cyt}]}{[P_{plant-cyt}]}\right) \quad \text{(eq. 24)}$$

$$\Delta V_0 = V_f^0 - V_p^0 \quad \text{(eq. 25)}$$

The final simplifications in equations (23) and (24) are justified by the fact that under normal conditions the cytosolic pH in the plant and fungal cytosol is buffered to $pH_{plant\text{ -}cyt} \approx pH_{fungus\text{-}cyt} \approx 7.0$.

For the parameters $x = A, B, D, E, G, K, [C_{plant\text{-}cyt}], [C_{fungus\text{-}cyt}], [P_{plant\text{-}cyt}], [P_{fungus\text{-}cyt}]$ the equations (20-22) are of three different types as specified in Table 1:

Type (1): $I = \frac{\alpha\cdot x}{x+\beta}$ $I(x{=}0){=}0$ and $I(x\rightarrow\infty)\rightarrow\alpha$ (e.g. H/C-flux, Fig.8A)

Type (2): $I = \frac{\delta\cdot x+\gamma}{x+\varepsilon}$ $I(x{=}0){=}\gamma/\varepsilon$ and $I(x\rightarrow\infty)\rightarrow\delta$ (e.g. H/P-flux, Fig.8B)

Type (3): $I = a\cdot ln(x) + b$

Type (1) and (2) curves saturate with very large x-values. Type (1) curves are zero for x=0, while type (2) curves do not reach the zero level. Curves of the same shape were also determined by computational parameter screening (Fig. 4; Fig. 8).

Table 1 Equation types for the different parameters.

	A	*B*	*D*	*E*	*G*	*K*	$[C_{plant\text{-}cyt}]$	$[C_{fungus\text{-}cyt}]$	$[P_{plant\text{-}cyt}]$	$[P_{fungus\text{-}cyt}]$
$I_{H/C}$	(1)	(2)	(1)	(2)	(2)	(2)	(3)	(3)	(3)	(3)
$I_{H/P}$	(2)	(1)	(2)	(1)	(2)	(2)	(3)	(3)	(3)	(3)
I_{BG}	(2)	(2)	(2)	(2)	(1)	(1)	(3)	(3)	(3)	(3)
